# First confirmed occurrence of the yellow fever virus and dengue virus vector *Aedes* (*Stegomyia*) *luteocephalus* (Newstead, 1907) in Mozambique

**DOI:** 10.1186/s13071-020-04217-9

**Published:** 2020-07-14

**Authors:** Ana Paula Abílio, Ayubo Kampango, Eliseu J. Armando, Eduardo S. Gudo, Luís C. B. das Neves, Ricardo Parreira, Mohsin Sidat, José M. Fafetine, António Paulo G. de Almeida

**Affiliations:** 1 Instituto Nacional de Saúde (INS), Maputo, Província de Maputo Mozambique; 2 Centro de Biotecnologia, Universidade Eduardo Mondlane (UEM), Maputo, Mozambique; 3 Direcção Provincial de Saúde de Niassa (DPSN), Lichinga, Mozambique; 4 Department of Veterinary Tropical Diseases, University of Pretoria (UP), Pretoria, South Africa; 5 GHTM, Institute of Hygiene and Tropical Medicine (IHMT), Universidade Nova de Lisboa, Lisboa, Portugal

**Keywords:** New record, Arthropod-borne, Virus, Vector, Aedine, Mosquito

## Abstract

**Background:**

Mozambique, same as many other tropical countries, is at high risk of arthropod-borne virus (arbovirus) diseases and recently two dengue virus (DENV) outbreaks occurred in the northern part of the country. The occurrence of some important vector species, such as *Aedes* (*Stegomyia*) *aegypti* (Linnaeus) and *Ae.* (*Stg.*) *albopictus* (Skuse), besides several other sylvatic vectors, have been reported in the country, which may indicate that the transmission of some arboviruses of public health importance may involve multiple-vector systems. Therefore, knowing the occurrence and distribution of existing and the new important vectors species, is crucial for devising systematic transmission surveillance and vector control approaches. The aim of this study was to map the occurrence and distribution of mosquito species with potential for transmitting arboviruses of human and veterinary relevance in Niassa Province, Northern Mozambique.

**Methods:**

Field entomological surveys were undertaken in April 2016 in Lago District, Niassa Province, northern Mozambique. Breeding sites of mosquitoes were inspected and immature stages were collected and reared into adult. Mosquitoes in the adult stages were morphologically identified using taxonomic keys. Morphological identification of *Aedes* (*Stegomyia*) *luteocephalus* (Newstead) were later confirmed using dissected male genitalia and molecular based on the phylogenetic analyses of the sequenced barcode (*cox*1 mtDNA) gene.

**Results:**

A total of 92 mosquito larvae collected developed into adults. Of these, 16 (17.39%) were morphologically identified as *Ae. luteocephalus*. The remaining specimens belonged to *Ae.* (*Stg.*) *aegypti* (*n* = 4, 4.35%), *Ae.* (*Aedimorphus*) *vittatus* (*n* = 24, 26.09%), *Anopheles garnhami* (*n* = 1, 1.09%), *Culex* (*Culiciomyia*) *nebulosus* (*n* = 28, 30.43%), *Eretmapodites subsimplicipes* (*n* = 18, 19.57%) and *Toxorhynchites brevipalpis* (*n* = 1, 1.09%), taxa already known to the country. Male genitalia and phylogenetic analyses confirmed the identity of *Ae. luteocephalus* specimens collected in this study.

**Conclusions:**

To our knowledge, this is the first detection of *Ae. luteocephalus* in Mozambican territory, a vector species of yellow fever virus (YFV), Zika virus (ZIKV) and dengue virus (DENV) in Africa. Further studies are encouraged to investigate the role of *Ae. luteocephalus* in the transmission of arboviral diseases in Mozambique.
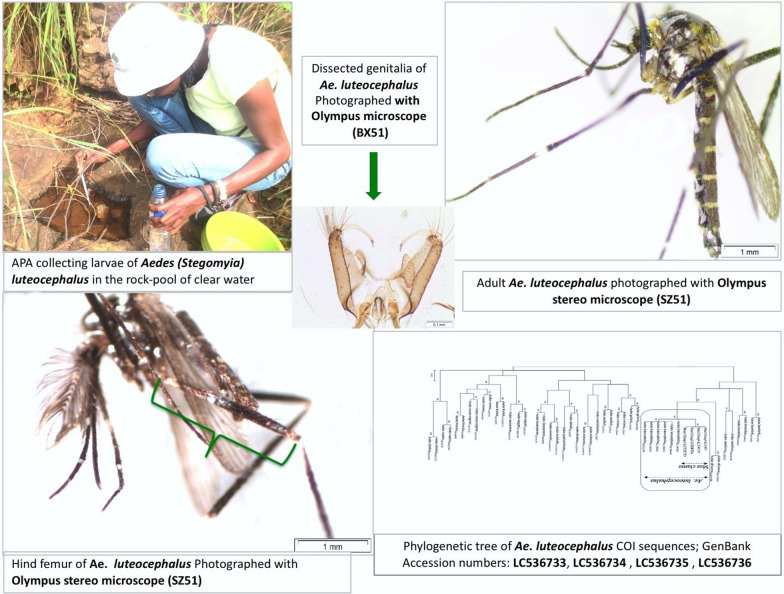

## Background

The occurrence and distribution of mosquito-borne arboviruses, particularly those transmitted by *Aedes* species, such as dengue (DENV), Zika (ZIKV) and yellow fever viruses (YFV) represent a serious threat to global health, particularly to African sub-Saharan countries [1–6]. It has been estimated that annually nearly 3,312,040 cases and 4,032 deaths of DENV infections occur worldwide [3, 7]. Zika virus, on the other hand, has been declared a public health emergency of international concern since 2016 [6]. Likewise, re-emergence of yellow fever cases has been lately observed in some African countries and Brazil, despite the existence of an effective vaccine that could protect populations and control the disease [8].

Mozambique is home to several mosquito species, some of which are widely known and suspected arbovirus vectors [6, 9–11]. However, the role of the country’s mosquito fauna in sustaining the transmission of endemic arboviral diseases such as dengue, yellow fever and chikungunya still remains poorly understood. One possible reason for such neglect may be due to the overwhelming number of malaria transmission cases that has been recorded throughout the country [12]. Most recently, two dengue virus outbreaks were observed in the northern region of Mozambique amounting to thousands of cases of infection [13, 14]. Likewise, the presence of major arbovirus vectors such as *Ae.* (*Stg.*) *aegypti* and *Ae.* (*Stg.*) *albopictus*, besides others with a more sylvatic distribution, has been reported in the country [6, 9], implying that arbovirus transmission dynamics may likely involve multiple-vector systems. These findings underscore the need for a thorough understanding of occurrence and arbovirus transmission role, of overlooked important potential vectors of public health importance. *Aedes* (*Stg.*) *luteocephalus* (henceforth, *Ae. luteocephalus*) is a mosquito species native to Africa, reported in *circa* twenty countries, particularly in the western and central regions of the continent, as well as in southern African countries such as Botswana and Zimbabwe [15–22]. This species has varied distribution throughout different geographical landscapes comprising forests, savannah, mangrove gallery, as well as intermediate landscapes between sylvatic and urban areas, where it has been found breeding in a diversity of natural and human-made larval sites [15, 17, 20].

*Aedes luteocephalus* is a competent vector for YFV [23] and can be an important vector of ZIKV and DENV, as observed in competence assays elsewhere in West and Central Africa [17, 20, 24]. Therefore, an in-depth understanding of the occurrence and distribution of these important vectors of arboviruses is crucial for devising accurate and effective evidence-based transmission control measures. The aim of this study was to map the occurrence and distribution of mosquito species with potential of transmitting arboviruses of human and veterinary relevance in the region.

Therefore, this report represents, to the best of our knowledge, the first confirmed record of *Ae. luteocephalus* in Mozambique. The implication of this discovery in the design of arthropod-borne virus (arbovirus) surveillance and control measures in Mozambique is briefly discussed.

## Methods

### Study site and sampling strategy

Entomological field surveys were conducted in April of 2016 in Lago District, neighborhood of Maniamba (12°41.881′S, 34°48.539′E), Niassa Province in northern Mozambique. All potential types of natural and artificial mosquito breeding sites were surveyed for the presence of mosquito immature stages. Mosquito larvae and pupae were sampled following standard operating procedures [25]. Additionally, used car tyres filled with water were placed for approximately 500 m apart in a transect along the main road crossing Chapama forest and Luaui River in an effort to collect as many samples as possible at different sites in the vicinity, to better sample the area. The tyres were left in the field for 8 days, after which they were surveyed for immature mosquitoes. Each breeding place was surveyed using a Pasteur pipette. Collected specimens were sorted, placed in the 500 ml plastic bottles, filled up to 75% of water from specific breeding place and labelled accordingly. All samples collected were then transported to local insectaries for rearing to adults [25, 26]. Preliminary morphological identification was conducted on adult stages emerged, using taxonomic keys [15, 21, 27–29]. Adult specimens were preserved individually in single 1.5 ml Eppendorf tubes at − 80 °C for further morphological and molecular analysis. Whole mosquitoes, male and female, of *Ae. luteocephalus* were re-observed and male terminalia were separated from the abdomen and adsorbed in Marc André solution [27]. Genitalia were dissected under stereomicroscope and mounted in formic acid-polyvinyl alcohol (PVA) solution between a slide and a cover slip [27, 28] and photographed under Olympus stereomicroscope SZ51 (Olympus, Seoul, South Korea), Olympus microscope (BX51, Olympus, Seoul, South Korea) and an Olympus SC30 digital camera (Olympus, Tokyo, Japan), respectively.

### Molecular analyses of adult mosquito specimens

Genomic DNA was extracted from remaining the abdomen and legs of 4 males, as described in Mixão et al. [27]. Molecular analysis was targeted at the barcoding section between positions 58 to 705 encoding the N-terminal section of the mitochondrial cytochrome *c* oxidase subunit 1 gene (*cox*1 mtDNA). Amplification of *cox*1 mtDNA was performed using LCO1490 and HCO2198 specific primers under PCR conditions as described by Folmer et al. [30]. The nucleotide (nt) sequences obtained were deposited in GenBank [31] under the accession numbers LC536733-LC536736).

The degree of correspondence between the barcode *cox*1 mtDNA gene sequences obtained in this study were compared against those at GenBank database using BLASTn, (https://blast.ncbi.nlm.nih.gov/Blast.cgi) and Barcode of Life Data Sytems-v4 (http://www.boldsystems.org/) [32].

Phylogenetic reconstructions using *cox*1 molecular data were carried out from multiple alignments of nt sequences obtained using the iterative G-INS-I method as implemented in MAFFT v. 7 [33]. Subsequently, attained sequences were edited using both GBlocks [34] and visual inspection using BioEdit 7.0.5 [35] to ensure the correct alignment of homologous codons. Phylogenetic analysis was carried out using the Maximum Likelihood (ML) optimization criterion and GTR + Γ + I (GTR-General Time Reversal, Γ-Gamma distribution, I-proportion of invariant sites) as the dataset best-fitting evolutionary model, as suggested by jModelTest2 [36]. The ML phylogenetic tree was constructed with W-IQ-tree [37], using the bootstrap test (with 1000 random data resampling’s) for assessment of the tree topological stability. The tree was edited with FigTree 1.4.4 (http://tree.bio.ed.ac.uk/software/figtree/). Due to the lack of a satisfactory number of examined sequences and collected specimens, we were unable to run haplotype network analysis for robust inference of the origin of *Ae. luteocephalus* collected in this study.

## Results and discussion

A total of 92 adult mosquitoes emerged from collected larvae and pupae; of these, 16 were tentatively identified as *Ae. luteocephalus* (12 females and 4 males) based on morphological features. The remaining specimens were identified as *Anopheles* (*Celia*) *garnhami* (*n* = 1), *Ae.* (*Aedimorphus*) *vittatus* (*n* = 24), *Ae.* (*Stg.*) *aegypti* (*n* = 4), *Culex* (*culiciomyia*) *nebulosus* (*n* = 28), *Eretmapodites subsimplicipes* (*n* = 18) and *Toxorhynchites brevipalpis* (*n* = 1) (Table 1).

**Table 1:** Date of collection, mosquito species, sex, their respective niches, percentage (%) and number of mosquitoes collected from Mozambique

Date of collection	Habitat	Species	Sex (*n*)	Subtotal (%)
1 April 2016	Rock-pool of clear water	*Aedes* (*Stegomyia*) *luteocephalus*	M (4)/F (12)	16 (17.39)
		*Aedes* (*Aedimorphus*) *vittatus*	M (5)	5 (5.43)
8 April 2016	Tyre placed as “ovitraps”	*Anopheles* (*Celia*) *garnhani*	M (1)	1 (1.09)
		*Aedes* (*Stegomyia*) *aegypti*	M (4)	4 (4.35)
		*Aedes* (*Aedimorphus*) *vittatus*	M (3)/F (16)	19 (20.65)
		*Culex* (C*uliciomyia*) *nebulosus*	M (10)/F (18)	28 (30.43)
		*Eretmapodites subcimplicipes*	M (3)/F (15)	18 (19.57)
		*Toxorhynchites brevipalpis*	F (1)	1 (1.09)
Total collected				92

*Abbreviations*: F, female; M, male

Ten females of *Ae. luteocephalus* collected in this survey were deposited in the insect depository of Instituto Nacional de Saúde (INS) in Maputo Province, Mozambique, stored in individual Eppendorf^®^ tubes (accession numbers MZ113-a1.2, a1.4–a1.12) and 6 specimens (2 females and 4 males) deposited in the Entomoteca (Insect collection) of the Institute of Hygiene and Tropical Medicine (IHMT), Lisbon, Portugal (accession numbers MZ113-a1.1–a1.3 and MZ113-a2.1–a2.4).

All 16 larvae, which gave rise to the adults *Ae. luteocephalus* and 5 *Ae. vittatus* were found cohabiting in a rock-pool of clear water, with approximately 20 × 15 cm (Additional file 1: Figure S1), located at the Luaui riverbank and exposed to sunlight. Other species including the remaining 19 *Ae. vittatus* were obtained from other breeding sites, namely the tyres that were placed as “ovitraps”, while no specimens of *Ae. luteocephalus* were obtained from any other breeding site (Table 1). Exhaustive studies are required to collect more information about *Ae. luteocephalus* distribution and infestation to better understand the importance of this and other arbovirus vectors throughout the country.

The habitat where they were found corresponds to its natural range of tropical forest habitats. Indeed, *Ae. luteocephalus* can be found in forests, savannah, mangrove gallery forest and also in intermediate landscapes between sylvatic and urban areas [15, 17, 20]. Bionomically the specie utilizes varied range of breeding places as rot holes, tree holes, rock holes, bamboos, bamboo stems, tree fork, plastic bottles and artificial containers in height up to 9 metres [15–20]. *Aedes luteocephalus* breeding sites with similar features have also been reported elsewhere and in association with *Ae. africanus*, of the same group, but not to our knowledge, with *Ae. vittatus*, which is not surprising, as this later species also favors rock pools [15–20].

Preliminary analysis of the nucleotide sequences of *cox*1 mtDNA obtained from the 4 males revealed completely identical sequences, which suggests that the *Ae*. *luteocephalus* larvae sequenced were siblings [28], possibly hatched from eggs laid by a single female, as mtDNA is maternally inherited, quite possible given the small dimensions of the breeding rock pool (Additional file 1: Figure S1).

Laboratory experiments have shown that *Ae. luteocephalus* can transmit yellow fever with an efficiency comparable to *Ae. aegypti* [23], readily bites humans and is involved in the transmission of YFV in West and Central Africa, and chikungunya virus (CHIKV), ZIKV and DENV2 have been isolated from it in West Africa [15]. Although *Ae. luteocephalus* habitat has been essentially rural and sylvatic, increasing demographical expansion and human pressure on forest resources, for logging and farming as it has been observed in the studied place. This condition might also increase the likelihood of vector-human and, therefore, the risk of rural arbovirus epidemics. Therefore, additional studies are urgently needed to investigate the effect of anthropogenic activity on arboviruses transmission risk in Lago District.

**Fig. 1:**
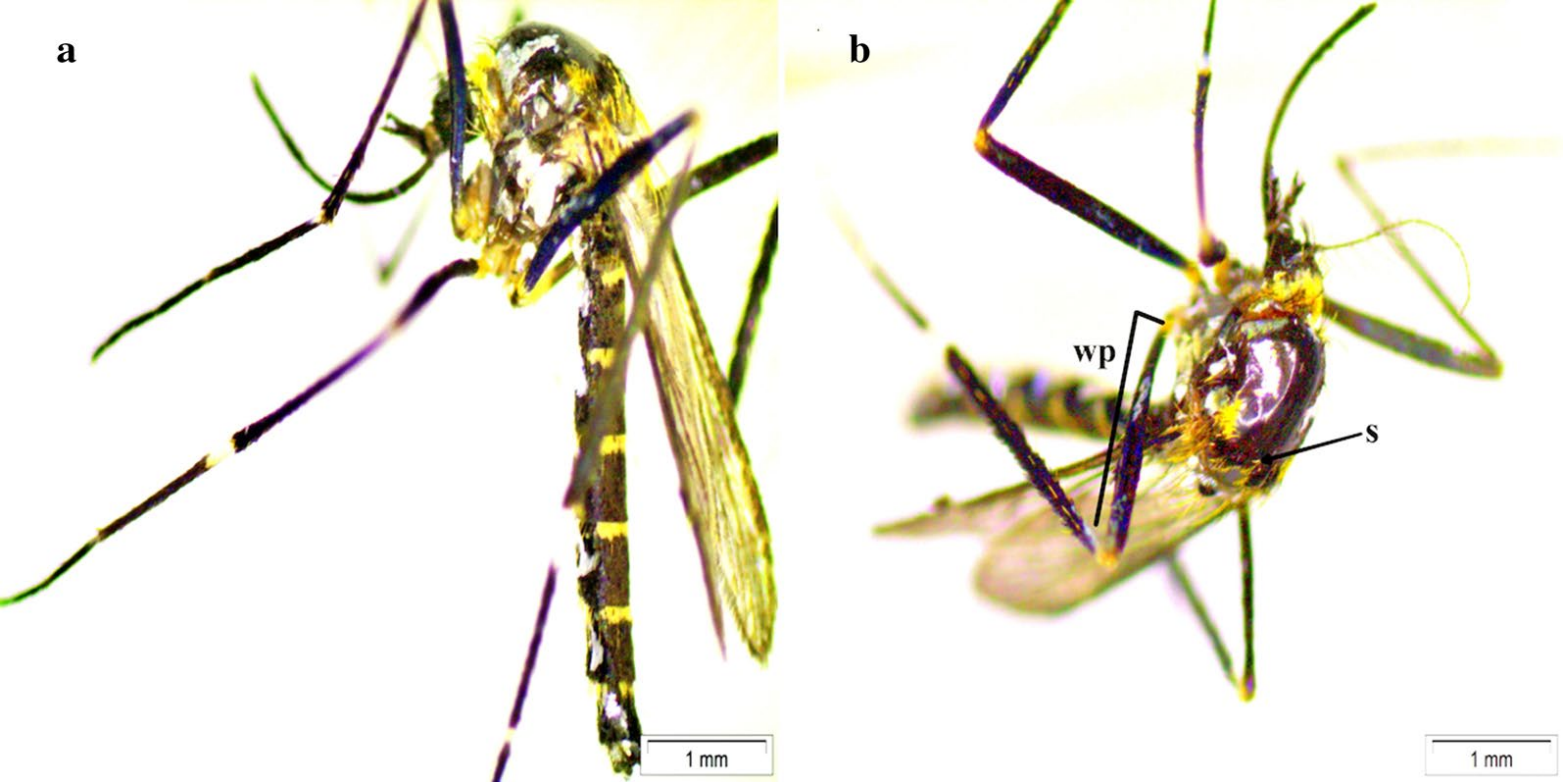
**a** Adult female of *Aedes luteocephalus* specimen showing general characters. **b** Adult specimen highlighting the main diagnostic features including for the scutum (with median-longitudinal yellow stripe) (s) and hind tarsomere anteriorly with large pale band at base and two large white patches on median and apical areas (wp), both images at 20× magnification

All *Ae. luteocephalus* specimens collected in this study, had a distinct middle longitudinal yellow stripe of thin scales in the scutum region; scutellum with wide white scales on lateral lobes; basal pale band on terga II-VI more yellow; and hind femur anteriorly with a huge light band at base and alongside two sizable white spots on median and apical regions (Fig. 1). These characteristics are similar to those described by Huang [15] and Jupp [21].

**Fig. 2:**
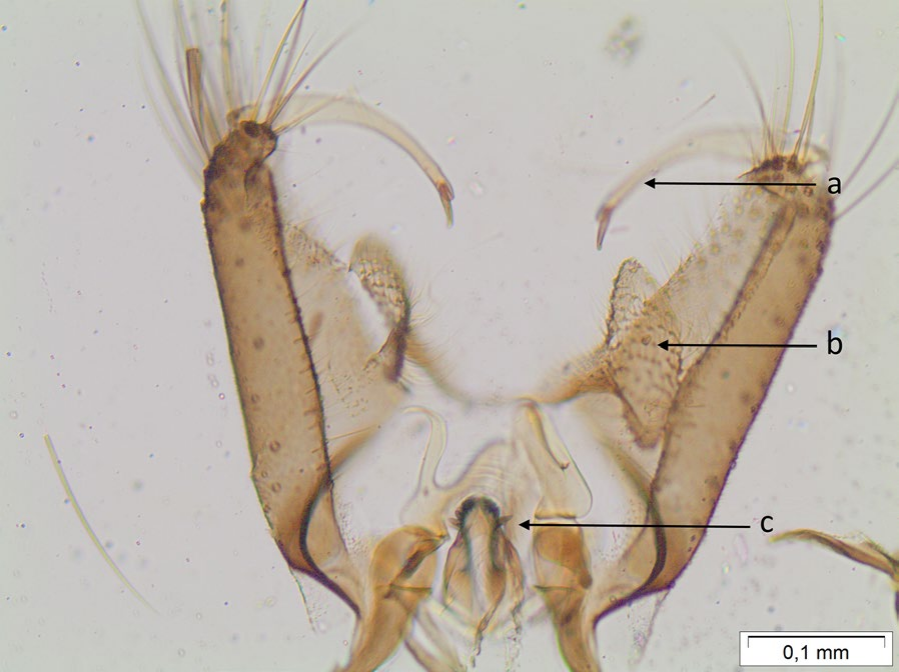
Dissected male genitalia of *Aedes luteocephalus* showing gonocoxites with gonostylus (**a**) with gonostylar claw and claspette (**b**) large, lobed with distal expanded portion, oval in dorsal view, with numerous simple setae on the apicolateral portion, and with some short setae on the apicomesal portion and aedeagus (**c**) at 100× magnification. *Scale-bar*: 100 µm

All four dissected male genitalia showed gonocoxites with gonostylus simple with few setae in the apical quarter and a long slender gonostylar claw, claspette large, lobed, with distal expanded portion, oval in dorsal view, with numerous simple setae on the apicolateral portion, and with some short setae on the apicomesal portion (Fig. 2). These are considered the most important distinctive features that separate the species from other members of the *africanus* group, namely, *Aedes* (*Stg.*) *africanus*, to which it belongs [15, 21].

**Fig. 3:**
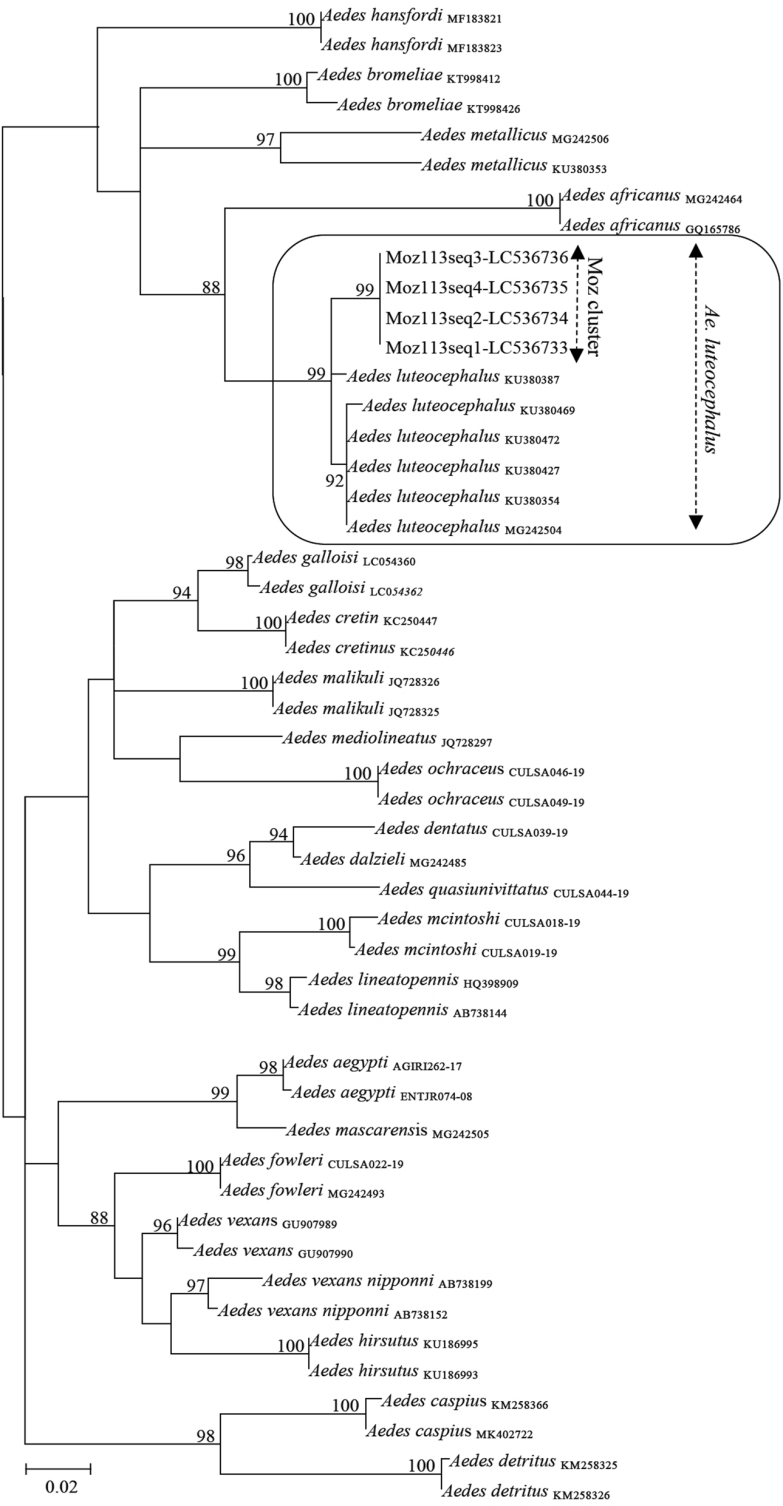
Maximum likelihood phylogenetic tree based on partial *Aedes cox*1 sequences. Nodal support values ≥ 75 are shown. The reference sequences used are indicated with either their GenBank accession number or BoldSystems code. The sequences generated in this study are indicated in bold by their laboratory code and accession numbers and are grouped in a monophyletic cluster indicated as Moz cluster. The scale-bar indicates the number of nucleotide substitutions per site

Barcode gene sequences of all specimens analyzed displayed 97.65–98.12% sequence identity with homologues using BLAST (MegaBlast option) and 97.82–98.26% identity in the BOLDSYSTEMS database with sequences of *Ae*. *luteocephalus* from Tanzania and Kenya, thereby confirming its taxonomic identity [19, 20, 22]. These findings represent, as far as we are aware, the first confirmed record of *Ae. luteocephalus* in Mozambique, while all other specimens collected in this survey correspond to taxa already known for the country. Additionally, phylogenetic reconstruction analysis carried out on the basis of a dataset of multiple *Aedes* species of the subgenera *Stegomyia*, *Aedimorphus*, *Neomelaniconion* and *Ochlerotatus*, clearly placed the *cox*1 sequences obtained in the course of this study in a topologically stable monophyletic cluster that only included *Ae. luteocephalus* reference sequences (Fig. 3). This further confirms the morphological, barcode and sequence similarity-based identifications presented above. Therefore, our results clearly confirm that *Ae. luteocephalus* collected in the study area are quite similar to those from the neighboring countries Tanzania and Kenya [19, 20, 22] and have now been found as part of a wide survey in the country for vectors of arboviruses Abílio et al., unpublished data). Further haplotype network analyses are recommended to ensure for robust inference of exact origin of *Ae. luteocephalus* from Mozambique.

## Conclusions

Comparative morphological, molecular and phylogenetic analyses have consistently shown, for the first time, the occurrence in Mozambican territory of *Ae. luteocephalus*, a competent vector of yellow fever virus and dengue fever virus in Africa. This finding may help fill the gaps of our knowledge about the distributional ecology of this important and overlooked arbovirus vector. Further field and laboratory surveys are encouraged to investigate the role of *Ae. luteocephalus* in the transmission of arboviruses in Mozambique.

## Supplementary information

**Additional file 1: Figure S1.** Corresponding author collecting larvae of *Ae. luteocephalus* in a rock-pool with clear water approximately 20 × 15 cm, located at the Luaui riverbank, Lago District, neighbourhood of Maniamba, Niassa Province, northern Mozambique.

## Data Availability

The datasets used and/or analyzed during the present study are available from the corresponding author upon reasonable request.
