# Supplementary figures and images for: First confirmed occurrence of the yellow fever virus and dengue virus vector Aedes (Stegomyia) luteocephalus (Newstead, 1907) in Mozambique
Source: Parasit Vectors. 2020 Jul 14;13:350. doi: 10.1186/s13071-020-04217-9 (PMC7537105; doi:10.1186/s13071-020-04217-9)

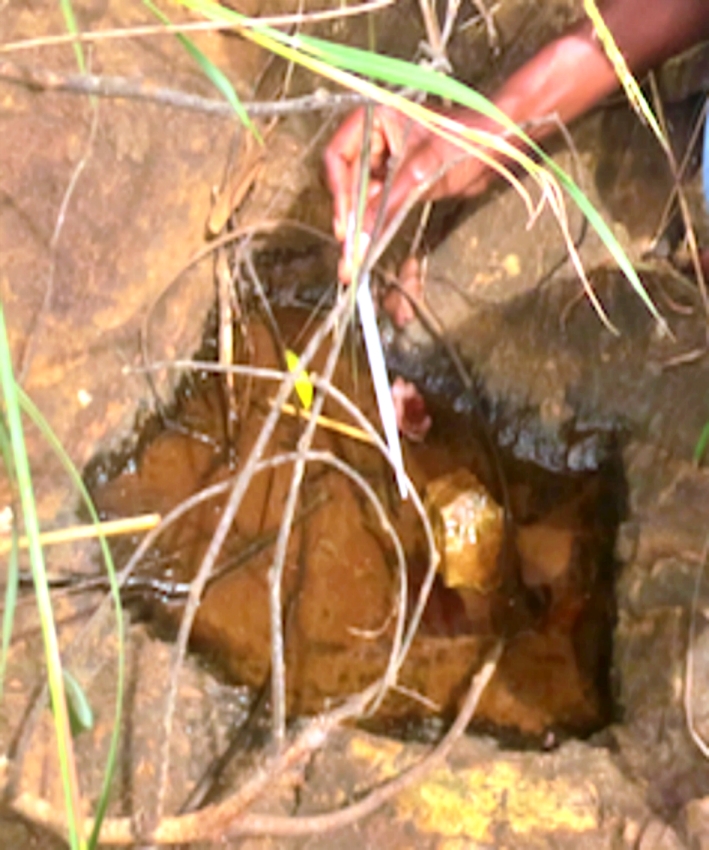

Supplement: Supplementary file 1 — Additional file 1: Figure S1. Corresponding author collecting larvae of Ae. luteocephalus in a rock-pool with clear water approximately 20 × 15 cm, located at the Luaui riverbank, Lago District, neighbourhood of Maniamba, Niassa Province, northern Mozambique. [file 13071_2020_4217_MOESM1_ESM.jpg]
